# The Current Situation and Future Trend of Leukemia Mortality by Sex and Area in China

**DOI:** 10.3389/fpubh.2020.598215

**Published:** 2020-12-11

**Authors:** Baojing Li, Hong Tang, Zilu Cheng, Yuxiao Zhang, Hao Xiang

**Affiliations:** ^1^Department of Global Health, School of Health Sciences, Wuhan University, Wuhan, China; ^2^Global Health Institute, Wuhan University, Wuhan, China; ^3^School of Chemistry, Chemical Engineering and Life Sciences, Wuhan University of Technology, Wuhan, China

**Keywords:** leukemia, mortality, joinpoint analysis, projection, SDGs

## Abstract

Leukemia is one of the most common cancers. We conducted this study to comprehensively analyze the temporal trends of leukemia mortality during 2003–2017 and project the trends until 2030. We extracted national-level data on annual leukemia mortality from China Health Statistics Yearbooks (2003–2017). We applied the Joinpoint regression model to assess leukemia mortality trends in urban and rural China by sex during 2003–2017. We also produced sex-specific leukemia mortality using the adjusted Global Burden Disease (GBD) 2016 projection model. In urban areas, age-standardized leukemia mortality decreased significantly among females during 2003–2017 (APC = −0.9%; 95% CI: −1.7, −0.1%). In rural areas, significant decreases of age-standardized leukemia mortality were both found among males (APC = −1.7%; 95% CI: −2.9, −0.5%) and females (APC = −1.6%; 95% CI: −2.6, −0.7%) from 2008 to 2017. Rural-urban and sex disparities of leukemia mortality will continue to exist until the year 2030. According to projection, the leukemia mortality rates of males and rural populations are higher than that of females and urban populations. In 2030, leukemia mortality is projected to decrease to 3.03/100,000 and 3.33/100,000 among the males in urban and rural areas, respectively. In females, leukemia mortality will decrease to 1.87/100,000 and 2.26/100,000 among urban and rural areas, respectively. Our study suggests that more precautionary measures to reduce leukemia mortality are need, and more attention should be paid to rural residents and males in primary prevention of leukemia in China.

## Introduction

Leukemia, also known as leukemia, is a group of blood cancers that start in blood-forming tissues and result in large numbers of abnormal blood cells in the bloodstream. The main types of leukemia include acute myeloid leukemia, acute lymphoblastic leukemia, chronic lymphocytic leukemia, chronic myelogenous leukemia, and hairy cell leukemia (1, 2). Leukemia ranks as the tenth most common cause of death from cancer worldwide with an estimated 309,006 deaths in 2018 (3). According to the Global Burden Disease (GBD) 2017 report, an estimated 141,317 new cases and 60,010 deaths of leukemia occurred in 2017 in China, accounting for 27.3% of 518,485 new cases and 17.3% of 347,583 deaths worldwide, respectively (4–6).

In the socioeconomic context of economic growth and urbanization, China has long been characterized by large rural-urban disparities in health care. Leukemia is closely related to ionizing radiation, benzene, formaldehyde, pesticides and infectious agents (7), and rural residents are more likely to expose to these risk factors, which could lead to the rise of leukemia mortality in rural areas and the enlarging rural-urban disparities for leukemia mortality. Besides, sex disparities are also observed in leukemia mortality. More men than women are diagnosed with leukemia and die from the disease. About 30% more men have developed leukemia than women (8). Thus, it is necessary to examine the trends of leukemia mortality by sex and area in China to provide positive implications for policy-makers in their attempt to reduce health inequities related to leukemia.

Literature analyzing the mortality trends of leukemia in China is scarce. Although previous studies have reported leukemia mortality trends in China, they either covered a small proportion of the Chinese population in some certain cities such as Tianjin (9) and Kunshan (10), or no updated data were provided (11). Large-scale nationwide epidemiological study of leukemia mortality trends with the latest data in China is needed. Therefore, we comprehensively examined the temporal trends of leukemia mortality during 2003–2017 and project the trends until the year 2030 using the most valid and updated data available in China. In addition, we examined rural-urban and sex disparities in leukemia mortality. Key findings reported in this paper will be critically important for policy-makers in China to develop appropriate and effective prevention and treatment of leukemia, which will further contribute to achieving “leaving no one behind” (a key SDGs pledge) (12).

## Materials and Methods

### Data Source

We extracted age-specific crude mortality rates of leukemia by year from China Health Statistics Yearbooks (2003–2017), which cover disease-specific mortality stratified by age and sex in urban and rural populations. The age-standardized mortality rates were calculated by way of the world standard population, and age-specific rates were calculated for age groups of 0–14, 15–34, 35–54, 55–74, and ≥75-years, respectively. According to the China Health Statistics Yearbooks, the urban areas include municipalities under the direct administration of central government and prefecture-level cities. The rural areas include counties and county-level cities, and township health centers and village clinics are included as well. China Health Statistics Yearbooks are annual informative publications that reflect the development of health care and residents' health status in China. The data are collected by the Center for Health Information and Statistics (CHIS) of China through national routine death reporting system and have been recognized as representative of common cancers in China (13, 14). The data from China Health Statistics Yearbooks is of high quality. Compared with data from China Health Statistics Yearbooks, the quality of cancer registry data in China needs to be improved. At present, the number of cancer registries in China is insufficient, and the coverage of the population is not wide (15).

### Joinpoint Regression Analysis

We applied the Joinpoint regression model to analyze the leukemia mortality trends during 2003–2017 in urban populations and rural populations by sex, respectively. Several straight-line segments were connected at the Joinpoints, where the slope of leukemia mortality trend significantly changed. We assessed the annual percent changes (APCs) and the average annual percent changes (AAPCs) in leukemia mortality to describe the temporal trends. Joinpoint Regression Program 4.8.0.1(https://surveillance.cancer.gov/joinpoint/) was downloaded from the website of the US National Cancer Institute. The software takes trend data (e.g., cancer mortality rates) and fits the simplest joinpoint model that the data allow. The user supplies the minimum and maximum number of joinpoints. The program starts with the minimum number of joinpoint (e.g., 0 joinpoints, which is a straight line) and tests whether more joinpoints are statistically significant and must be added to the model (up to two points). This enables the user to test that an apparent change in trend is statistically significant. The tests of significance use a Monte Carlo Permutation method. The models may incorporate estimated variation for each point or use a Poisson model of variation. In addition, the models may also be linear on the log of the response (e.g., for calculating annual percentage rate change). The software also allows viewing one graph for each joinpoint model, from the model with the minimum number of joinpoints to the model with maximum number of joinpoints and the user could choose to present the software recommended model (16). All tests were 2-sided and the statistical significance was set at *p* < 0.05.

### Projection of Leukemia Mortality

We explored adjusted GBD method to project leukemia mortality. The GBD methodology considers the historical trend and weights more on recent changes, and was designed to use different methods to produce reasonable outcomes according to different types of data, which shows comparative reliability in projecting results. For instance, it will not produce a percentage below 0 or above 100% (17). We further adjusted the GBD methods (details below) and a calculation tool was designed to do the projections.

The adjusted GBD methods following the original GBD methodology to first convert data into logit-space (for percentage data) or natural-log space (for other data) and calculate the annual rate of change. Afterwards, a time-based weight matrix is established to add more weight to rate of change in recent years. The weight of rate of change in year t is:

(1) $$\begin{array}{r} {Weight}_{t}=\;\frac{{\left(t-1990\right)}^{\omega }}{\sum_{i=1991}^{T}{\left(i-1990\right)}^{\omega }} \end{array}$$

Where *T* is the last year with available data. The parameter ω was determined with a validity test: we selected the ω that used the first half of available data to predict the second half most accurately.

## Results

### Joinpoint Regression Analysis

**Figure 1:**
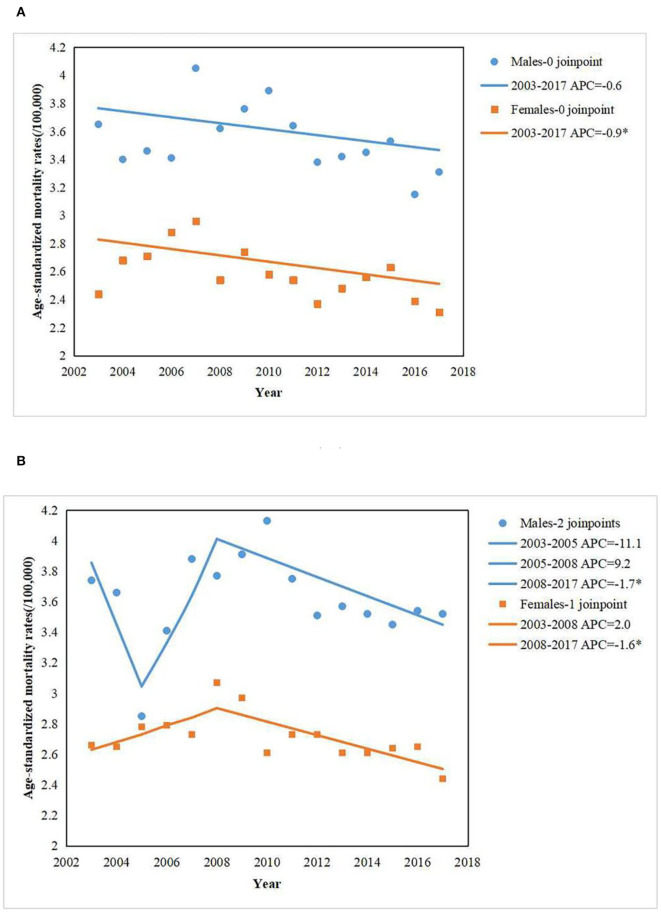
Age-standardized leukemia mortality (/100,000) in Chinese populations among urban **(A)** and rural areas **(B)** from 2003 to 2017. The scattered data points represent the leukemia mortality. The trend lines were estimate d by modeling leukemia mortality from Joinpoint analysis.

Figure 1A showed the trends of age-standardized leukemia mortality rates in urban areas, by sex from 2003 to 2017. In males, there was a non-significant decrease in age-standardized leukemia mortality rates from 2003 to 2017 (APC = −0.6%; 95% CI: −1.4, 0.2%). In females, a statistically significant decrease was presented from 2003 to 2017 (APC = −0.9%, 95% CI: −1.7, −0.1%). Figure 1B showed the trends of age-standardized leukemia mortality rates in rural areas, by sex from 2003 to 2017. In males, a statistically significant decrease was presented from 2008 to 2017 (APC = −1.7%; 95% CI: −2.9, −0.5%). In females, these was a non-significant increase in age-standardized leukemia mortality rates from 2003 to 2008 (APC = 2.0%; 95% CI: −0.4, 4.4%), followed by a statistically significant decrease from 2008 to 2017 (APC = −1.6%; 95% CI: −2.6, −0.7%). Sex disparities were substantial for leukemia mortality among urban and rural areas. Higher leukemia mortality rates were observed among men than among women during 2003–2017 (Figures 1A,B).

Table 1 presented the APCs and AAPCs of age-specific leukemia mortality (2003–2017). During 2003–2017, a significant decrease of age-specific leukemia mortality rates was presented in urban populations aged 15–34 years. However, an upward trend of age-specific leukemia mortality was presented in rural populations aged over 75 years during 2003–2009. In addition, leukemia mortality has a statistically significant increase in rural males aged 55–74 years during 2003–2017.

**Table 1:** Joinpoint regression analysis of age-specific and age-standardized leukemia mortality rates by sex and area during 2003–2017.

**Age**	**Urban areas**				**Rural areas**			
	**Males**		**Females**		**Males**		**Females**	
	**Period**	**APC (95% CI)**	**Period**	**APC (95% CI)**	**Period**	**APC (95% CI)**	**Period**	**APC (95% CI)**
0–14	2003–2017	−0.9 (−2.8, 0.9)	2003–2017	−0.8 (−2.4, 0.9)	2003–2017	−2.3 (−4.5, −0.1)^*^	2003–2017	−1.0 (−2.4, 0.5)
15–34	2003–2017	−1.5 (−2.7, −0.4) ^*^	2003–2017	−1.8 (−3.4, −0.1)^*^	2003–2017	−0.6 (−2.1, 0.9)	2003–2017	−2.1 (−3.6, −0.5)^*^
35–54	2003–2017	−2.9 (−3.9, −1.8) ^*^	2003–2005	16.1 (4.6, 29.0)^*^	2003–2017	−0.8 (−2.5, 0.8)	2003–2017	−2.0 (−3.8, −0.2)^*^
			2005–2017	−3.9 (−4.5, −3.3)^*^				
			AAPC	−1.3 (−2.6,0.1)				
55–74	2003–2017	0.6 (−0.8, 2.0)	2003–2017	−0.0 (−1.3, 1.3)	2003–2017	2.0 (0.3, 3.7)^*^	2003–2017	1.5 (−0.1, 3.1)
≥75	2003–2017	1.7 (−0.8, 4.3)	2003–2017	1.1 (−1.3, 3.5)	2003–2009	8.0 (3.6, 12.6)^*^	2003–2009	15.1 (6.7, 24.2)^*^
					2009–2017	−0.3 (−3.0, 2.4)	2009–2017	−3.3 (−8.0, 1.5)
					AAPC	3.2 (1.1, 5.3)^*^	AAPC	4.2 (0.3, 8.2)^*^
Age standardized Overall	2003–2017	−0.6 (−1.4, 0.2)	2003–2017	−0.9 (−1.7, −0.1)^*^	2003–2005	−11.1 (−22.2, 1.4)	2003–2008	2.0 (−0.4, 4.4)
					2005–2008	9.2 (−4.3, 24.7)	2008–2017	−1.6 (−2.6, −0.7)^*^
					2008–2017	−1.7 (−2.9, −0.5)^*^	AAPC	−0.4 (−1.3, 0.6)
					AAPC	−0.9 (−3.7, 2.0)		

*AAPC (average annual percent change) presented for full period*.

*APC, annual percent change; CI, confidence interval*.

* *Statistically significant trend (p < 0.05)*.

### Projection of Leukemia Mortality

**Figure 2:**
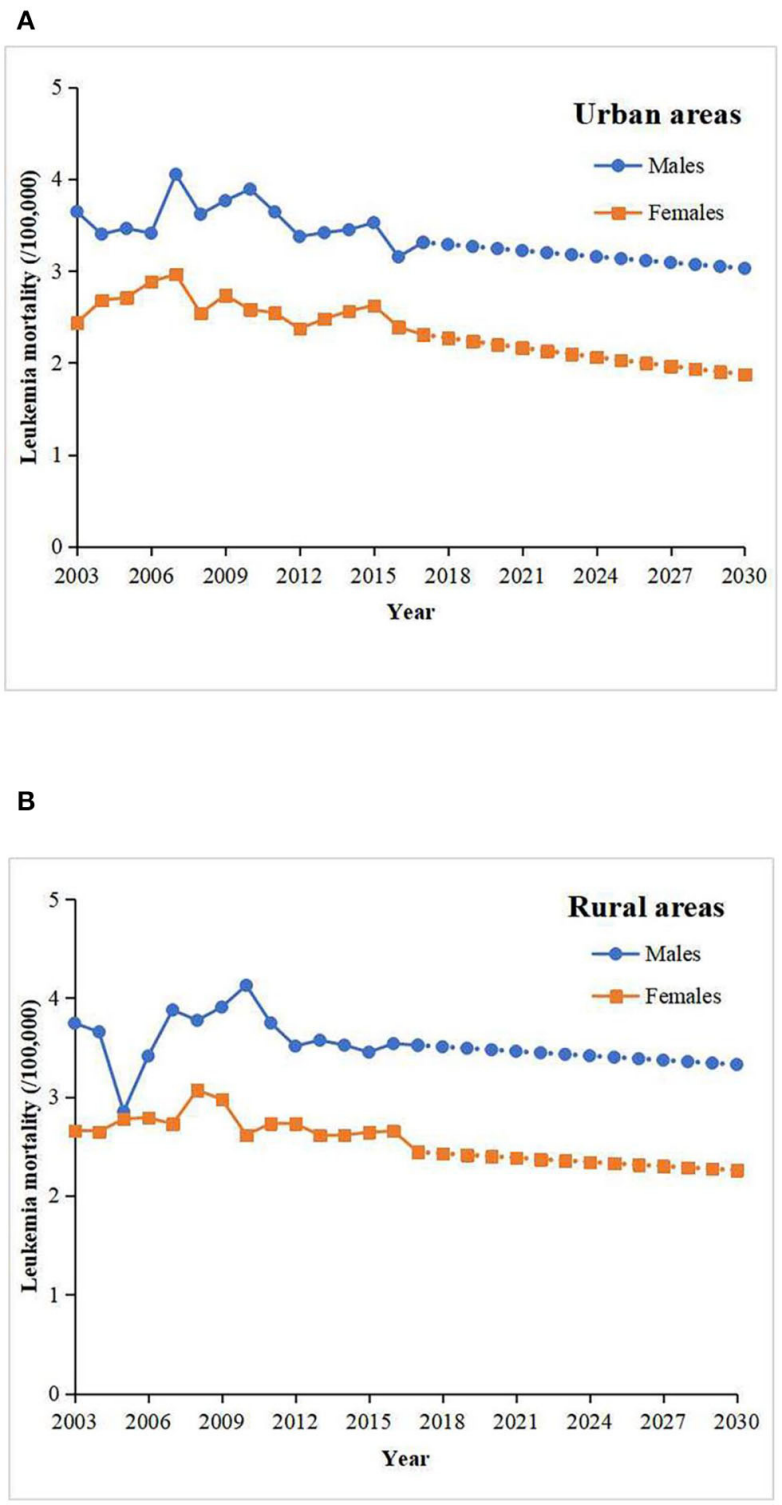
The projected leukemia mortality (/100,000) in the males and females among urban **(A)** and rural areas **(B)** from 2017 to 2030.

Sex and rural-urban disparities will still exist in leukemia mortality until the year 2030. Based on the projections of the leukemia mortality, it is estimated that among the males and females, leukemia mortality will decrease in both urban and rural areas (Figures 2A,B). In 2030, leukemia mortality is projected to drop to 3.03/100,000 in urban areas and 3.33/100,000 in rural areas among the males (Table 2). Among the females, leukemia mortality will continue to decrease in both urban and rural areas (Figures 2A,B), which will decrease to 1.87/100,000 and 2.26/100,000 among urban and rural areas in 2030, respectively (Table 2). It is also projected that the male population will continue to have higher leukemia mortality rates than the female population in both urban and rural areas (Figures 2A,B).

**Table 2:** Predicted number of leukemia mortality (/100,000) in males and females among urban and rural areas in 2020, 2025, and 2030.

**Population**	**Leukemia mortality (/100,000)**		
	**2020**	**2025**	**2030**
**Males**
Urban areas	3.24	3.13	3.03
Rural areas	3.47	3.40	3.33
**Females**
Urban areas	2.20	2.03	1.87
Rural areas	2.40	2.33	2.26

## Discussion

This study is an updated systematic analysis and projection of nationwide leukemia mortality rates in urban and rural China by using the latest representative data. From 2008 to 2017, leukemia mortality decreased among the males and females in urban and rural areas. In rural areas, leukemia mortality significantly decreased by 1.7 and 1.6% among the males and females, respectively, from 2008 to 2017. In urban areas, leukemia mortality significantly decreased by 0.9% in the females during 2003–2017. Rural-urban disparities and sex disparities were substantial for leukemia mortality, which will still exist until 2030. In 2030, leukemia mortality is projected to drop to 3.03/100,000 in urban areas and 3.33/100,000 in rural areas among the males. Among the females, leukemia mortality will decrease to 1.87/100,000 and 2.26/100,000 among urban and rural areas in 2030, respectively.

The findings on leukemia mortality trends are consistent with a previous study (9) in Tianjin, China from 1999 to 2015, which reported that the age-adjusted leukemia mortality among urban residents showed a decreasing trend with the APC of −1.12% (*p* = 0.026). Besides, significant drops in the mortality of adult patients diagnosed with myeloid leukemia were observed from 1994 to 2001 with the APC of −21.22% and from 1994 to 2003 with the APC of −12.86% for males and females, respectively, in southeastern Brazil (18). Mortality from leukemia steadily declined in the European Union with the APC of −3.7% in males and −3.8% in females at age 0–14, −2% in both sexes at age 15–44, and −0.6% in males and −1% in females at middle-age and overall from 1970 to 2009 (19). In Croatia, mortality rates were stable for myeloid leukemia in both sexes during 1988–2009 (20). These differences in trends were attributable to data source, research period, population composition, different types of leukemia involved in research, and different socioeconomic statuses of different countries.

Rural-urban disparities and sex disparities were substantial for leukemia mortality. An investigation in Gansu province of China found that the leukemia mortality in rural areas was 5.07 times higher than urban areas, with the mortality rates of 4.57/100,000 in rural areas and 0.90/100,000 in urban areas, respectively (21). A study conducted in the USA showed that the age-adjusted mortality rates per 100,000 population was 7.90 for metropolitan and 8.25 for nonmetropolitan areas during 1990–1992, and was 7.01 for metropolitan and 7.47 for nonmetropolitan areas during 2005–2009, respectively. This presented widening rural-urban disparities in leukemia mortality in the USA over time, which was contributed by the larger mortality reductions in metropolitan residents than non-metropolitan residents (22). Besides, another study in the USA depicted the estimated leukemia deaths by sex in 2019, which was 13,150 in men and 9,690 in women, respectively (23). A study in the European Union reported the age-standardized mortality rates of leukemia was 4.57/100,000 in males and 2.78/100,000 in females in 2011 (24). A study in Korea showed the crude leukemia mortality rates was 3.9 in men and 2.8 in women per 100,000 people in 2015 (25). All above studies suggested rural-urban and/or sex disparities existed in leukemia mortality in either high-income countries or low and middle-income countries.

There are several risk factors related to leukemia incidence, especially in rural male residents, and reductions in incidence can largely contribute to reductions in mortality. Ionizing radiation can be closely linked to the increase of leukemia mortality (26, 27). Several systematic reviews have presented a significant correlation between exposure to benzene and the incidence of acute myeloid leukemia (28, 29). Benzene is widely used as a solvent in industrial processes such as production of chemicals and plastics as well as oil processing. Tobacco smoke was also identified as one of the main potential sources of benzene exposure (30, 31). Use of tobacco is associated with a small increase in the risk of developing acute myeloid leukemia in adults (32). In addition, a statistically significant excess risk of leukemia has been observed among those occupationally exposed to formaldehyde in a meta-analysis (33). Besides, a few studies have recorded statistically significant excess risk of leukemia among agricultural workers exposed to pesticides (34–36). Moreover, there is an association of the increase of leukemia risk with exposure to infectious agents, which could be highly exposed to specific populations working on agriculture, animal husbandry and food processing (37–39). Rural residents, especially men, earn a living in farms and factories, where they may get exposed to ionizing radiation, benzene, formaldehyde, pesticides and/or infectious agents, and male population accounts for a large proportion of tobacco consumption. On the other hand, the improvement of early diagnosis technology for leukemia has provided effective help for the early detection of leukemia patients, which may lead to a decline in leukemia mortality to a certain extent. Chen et al. conducted a metabolomics study on the serum of new-onset Acute Myeloid Leukemia patients and healthy volunteers and found that six significantly different metabolites can be used for early diagnosis and prognostic analysis of Acute Myeloid Leukemia (40). Wang et al. found that the small molecule metabolites detected by the metabolomics method based on hydrogen spectrum nuclear magnetic resonance (^1^H-NMR) can be used for the early diagnosis of leukemia and the judgment of the severity of leukemia (41). In China, progress in the treatment of leukemia also contributed to the decline in leukemia mortality. Wang et al. systematically reviewed the new developments in clinical treatment of leukemia in China (42). In addition to conventional surgical treatment, the use of treatments with Chinese characteristics have also significantly improved the survival rate of leukemia patients. A meta-analysis of Chinese studies showed that the treatment of Acute Myeloid Leukemia with Homoharringtonine has a higher total complete response (CR) rate (43). Furthermore, new successful therapies will first be introduced in university hospitals or research institutes in urban areas and it will take some time until they are also applied in more peripheral hospitals in rural areas. So, their effect on mortality rates will take some time causing downward trends in rural areas. Consequently, the rural-urban disparities and sex disparities for leukemia mortality become substantial.

Rural-urban disparities and sex disparities will still exist in leukemia mortality until the year 2030. According to projection, the leukemia mortality rates of males and rural populations are higher than that of females and urban populations. In general, primary prevention is of particular relevance for cancer, where reductions in mortality are largely achieved through reductions in incidence (44). Thus, precautionary measures through health promotion and education are of paramount importance (45, 46). Primary prevention of leukemia can be accomplished in two ways: (i) avoiding or reducing the introduction of carcinogenic agents into the environment. For example, there is an urgent need to focus on health policies and systems, including pricing of tobacco (47), setting permissible exposure limits of ionizing radiation, benzene and formaldehyde in the workplace. What is more important is to earnestly ensure the implementation of the relevant policies and constantly improve the health systems (48). (ii) eliminating or reducing the exposure to carcinogenic agents that are already in our environment. Health education of high-risk individuals and populations, particularly in the early stages, should be taken into consideration. For instance, pesticides safety education and pesticide applicator regulation should be well designed to protect the public from pesticide misuse. In the long-run, accelerating economic growth in rural areas and improving living standards of rural residents could reduce their exposure to the risk factors, which is the fundamental solution to reduce rural-urban disparities in leukemia mortality. In the future, more attention should be paid to epidemiological evidence that is suggestive of an exposure-leukemia association, and experimental evidence of carcinogenicity supported by mechanistic considerations (44). Follow-up studies designed to determine whether leukemia mortality rates declined as the result of preventive measures (“before-after” effect) are in need.

There are several limitations in this study. First, leukemia is one of the most common cancers (49), and there are five main types of leukemia (2). Thus, further epidemiological studies of leukemia focusing on only one type of leukemia are needed. Second, the projection methodology, though carefully selected through comprehensive comparisons, can only reflect future changes based on historical trajectories. It fails to capture other possible changes such as stronger political commitment, new and effective technology advances and interventions, and does not consider the possibility of a ceiling effect. Moreover, we only roughly projected the leukemia mortality trends on the basis of past trends and do not model demographic, socioeconomic and other related factors into the model. Therefore, our results in the present study on temporal trends and projections of leukemia should be treated carefully.

## Conclusions

From 2008 to 2017, leukemia mortality decreased among the males and females in urban and rural areas. Rural-urban disparities and sex disparities were substantial for leukemia mortality, which will still exist until 2030. Precautionary measures should be taken, especially for rural residents and males. Studies focusing on epidemiological evidence, experimental evidence and “before-after” effect are needed to further explore the risk factors and prevention measures of leukemia.

## Data Availability Statement

The datasets presented in this study can be found in online repositories. The names of the repository/repositories and accession number(s) can be found below: http://www.cnki.net/.

## Author Contributions

HX: conceptualization, funding acquisition, project administration, writing—review, and editing. ZC and YZ: data curation and visualization. BL and HT: formal analysis, investigation, methodology, resources, software, validation, and writing—original draft. YZ and HX: supervision. All authors contributed to the article and approved the submitted version.

## Conflict of Interest

The authors declare that the research was conducted in the absence of any commercial or financial relationships that could be construed as a potential conflict of interest.
